# Hidden association of Cowden syndrome, PTEN mutation and meningioma frequency

**DOI:** 10.18632/oncoscience.305

**Published:** 2016-06-30

**Authors:** Eduard Yakubov, Ali Ghoochani, Rolf Buslei, Michael Buchfelder, Ilker Y. Eyüpoglu, Nicolai Savaskan

**Affiliations:** ^1^ Translational Neurooncology Laboratory, Department of Neurosurgery, Universitätsklinikum Erlangen, Friedrich-Alexander University (FAU) Erlangen-Nürnberg, Erlangen, Germany; ^2^ Department of Neurosurgery, Klinikum Nürnberg, Paracelsus Medical University, Nürnberg, Germany; ^3^ Department of Neuropathology, Universitätsklinikum Erlangen, Friedrich-Alexander University (FAU) Erlangen-Nürnberg, Erlangen, Germany; ^4^ BiMECON Ent., Berlin, Germany

**Keywords:** Cowden syndrome, PTEN gene, meningioma, multiple hamartoma syndrome, brain tumor

## Abstract

Cowden syndrome (CS) is clinically presented by multiple hamartomas, often with mucocutaneous lesions, goiter, breast cancer and gastrointestinal polyps. CS is a genetic disorder of autosomal dominant inheritance and is one distinct syndrome of the phosphatase and tensin homolog on chromosome 10 (PTEN) hamartoma tumor spectrum. Noteworthy, PTEN germline mutations are related to a wide range of brain tumors. We performed a systematic analysis and review of the medical literature for Cowden syndrome and meningioma and additionally present the case of a 29-year- old CS patient diagnosed with multiple meningiomas. We found strong evidence for high incidence of brain tumors in CS patients. In particular meningiomas and gangliocytomas/Lhermitte-Duclos disease were often associated with 8% and 9% respectively in CS patients. Since aberrations in chromosome 10q are associated with meningiomas, it is likely that the underlying mutations in CS drive to a certain extent neoplastic meningioma growth. We propose to include meningiomas and brain tumors in the major criteria spectrum of CS-related disorders. This could warrant early diagnosis of brain lesions and close therapy, as well as better monitoring of patients with CS.

## INTRODUCTION

Cowden syndrome (CS) is a clinically complex disease that is characterized by multiple hamartomas of ectodermal, mesodermal, and endodermal origin [1]. CS is now well recognized as a highly variable, autosomal-dominant hereditary cancer susceptible syndrome with increased risk of developing benign and malignant transformations [1-3].

In 1997, CS has first been linked to pathogenic mutations in the phosphatase and tensin homologue deleted on chromosome ten (PTEN) gene, located on chromosome 10q23.31 [4-6]. It is estimated to affect one in 200,000 individuals with a strong female predominance [7]. However, the incidence of CS before the identification of the underlying PTEN gene mutation was estimated to be 1:1,000,000 [8]. Approximately two-thirds of the mutations in CS occur in exons 5, 7, and 8 of the PTEN gene. Exon 5, which encodes the PTEN phosphatase core motif, comprises merely 20% of the PTEN gene. This domaine is also associated with approximately 40% of the identified mutations in CS [9]. Furthermore, PTEN encodes a dual phosphatase protein that negatively regulates the PI3K-Akt-mTOR pathway.

Given the importance of PTEN as tumor suppressor gene, mutations in the germline of PTEN have been linked to at least four distinct autosomal dominant syndromes including Cowden syndrome, Bannayan-Riley-Ruvalcaba syndrome, Proteus and Proteus-like syndrome. In addition, PTEN has also been found in various sporadic human cancers affecting the brain, breast, colon, thyreoid and endometrium [10].

Due to its variable clinical presentation, the diagnosis of Cowden Syndrome is still a clinical challenge. In practice, CS comes along with complex phenotypes and various clinical diagnoses which do not necessarily adjunct to genetic testing. However, technical standards have been established in managing PTEN screenings in individuals, such as the Cleveland Clinic adult clinical scoring system. The International Cowden Consortium has also contributed with a list of criteria to assess the diagnosis of Cowden syndrome. These criteria are based on the most common arising clinical features of this disease [11]. In fact, meningiomas and brain tumors are not included in the CS consortium criteria, presumbly due to the lack of data regarding its association with Cowden syndrome. Further, the screening recommendations of the National Comprehensive Cancer Network (NCCN) add help to diagnosing CS. Nevertheless, currently no CS diagnosis criteria provide specific guidelines for cerebral magnetic resonance imaging [11].

In this paper, we addressed the question whether the PTEN-associated CS appears with increased brain tumor incidence. We found strong evidence for the association between CS and the occurance of meningiomas.

## RESULTS

We performed a systematic meta-analysis of meningioma cases in patients suffering from CS (Table 1).

**Table 1:** PTEN mutational frequency in patients with Cowden syndrome

Tumor	Tumor Frequency	Cases count n=109
Breast cancer	37.61%	40
Thyroid cancer	14.68%	16
Endometrial cancer	10.09%	11
Colorectal cancer	9.17%	10
Renal cancer	3.67%	3
**Brain tumor:**	**20.18%**	21
*Gangliocytoma*	9.17%	10
***Meningioma***	**8.25%**	9
*Other brain tumors*	5.5%	5

Cowden syndrome with PTEN mutation detected in different tumor types. The tumor frequencies are summarized based on currently available peer-reviewed data. The extended quotation of references and clinical details is given in Supplementary Table S1.

Our study was based on 109 patients with CS and with confirmed PTEN mutation. We focused on cases of CS with PTEN mutation because, as mentioned above, PTEN is suspected to be a meningioma tumor suppressor gene due to its location on chromosome 10 [12-17]. According to the fact that CS is prevalent in females, the ratio of female to male in our study was 2.1 : 1 (n=107). The median age of the patients with CS was 43.5 years (n=101). Furthermore, 36 of the 109 patients did not have any malignant neoplasy or brain tumor. The most frequent cancers that we dealed with were breast, thyroid, endometrial, colorectal, and renal cancer as well as brain tumors, inter alia, gangliocytoma/ Lhermitte-Duclos disease and meningiomas (Table 1). Our results showed the following incidences in CS: breast cancer (37.61%), brain tumor (20.18%), thyroid cancer (14.68%), endometrial cancer (10.09%), colorectal cancer (9.17%), and renal cancer (3.67%). One fifth of patients with CS suffered from brain tumors, making this the second most frequent among the other cancers. The most common tumor in CS patients is breast cancer with nearly 38% of all cases. Within brain tumors, specially meningiomas were highly frequent in CS as confirmed by an incidence of 8.25% of patients with CS.

Although gangliocytomas (9.17%) are the most frequent brain tumors, the incidence of meningiomas is close to the incidence of gangliocytomas making up the majority of brain tumors in CS (Table 1). Astonishingly, males were more often affected with meningiomas as females (ratio of female to male was 4 : 5). This sex distribution is inverted to the general incidence of meningiomas in females and males (2.1:1). Since the International Cowden Consortium diagnostic criteria were basically drafted two decades ago where diagnostic criteria and imaging techniques were not standardized or simply did not exist, we propose to revise the criteria including menigiomas (Table 2).

**Table 2:** Revised Clinical Diagnostic Criteria for Cowden syndrome

***Major Criteria:*** Breast cancer Endometrial cancer Follicular thyroid cancer Lhermitte–Duclos disease (LDD) (Gangliocytoma) Meningioma GI hamartomas or ganglioneuromas Macrocephaly (≥97 percentile: 58 cm for females, 60 cm for males) Macular pigmentation of the glans penis Multiple mucocutaneous lesions: Trichilemmomas (≥3, at least one biopsy proven) Acral keratoses Mucocutaneous neuromas (≥3) Oral papillomas	***Minor Criteria:*** Autism spectrum disorder Colon cancer Esophageal glycogenic acanthosis (≥3) Lipomas (≥ 3) Intellectual disability (ie, IQ ≤ 75) Renal cell carcinoma Testicular lipomatosis Papillary thyroid cancer (papillary or follicular variant) Thyroid structural lesions (eg, adenoma, nodule(s), goiter) Vascular anomalies (including multiple intracranial developmental venous anomalies)
**Operational diagnosis in an individual (either of the following):** Three or more major criteria, but one must include macrocephaly, LDD, or GI hamartomas; or Two major and three minor criteria.	
**Operational diagnosis in a family where one individual is diagnostic for Cowden syndrome:** Any two major criteria with or without minor criteria; or One major and two minor criteria; or Three minor criteria.	

International Cowden Syndrome Consortium operational criteria for the diagnosis of Cowden syndrome (in the version of the year 2015) with the addition of meningioma as a major criteria (indicated in red letters).

### Case

**Figure 1:**
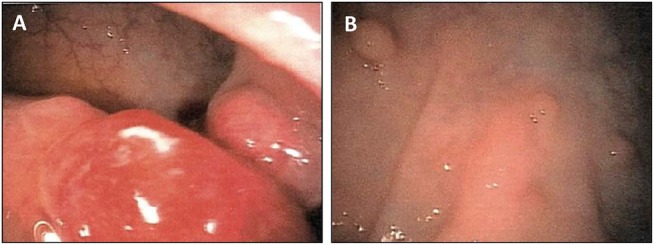
Clinical aspects of intestinal polyps in a patient suffering from Cowden syndrome **A.** Endoscopic aspects of two rectal tumors next to multiple polyps. **B.** Numerous intestinal polyps at the recto-sigmoid transition.

**Figure 2:**
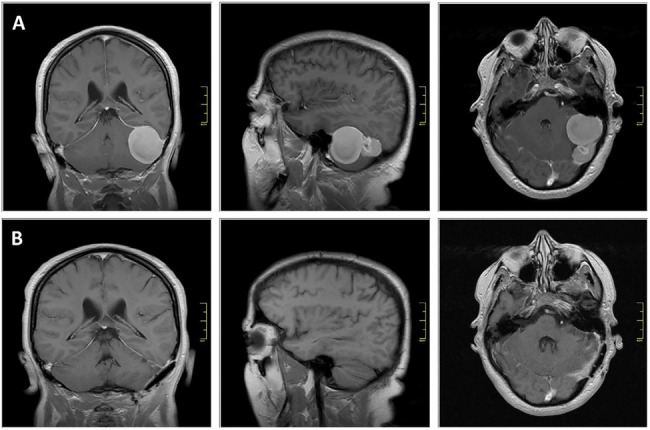
Meningioma in a patient with Cowden syndrome **A.** Coronal (left), sagittal (middle) and axial (right) gadolinium- enhanced T1-weighted MR images at pre-operative stage. **B.** contrast enhanced T1-weighted MRi images following neurosurgical intervention. Scale bar represents 4 cm.

**Figure 3:**
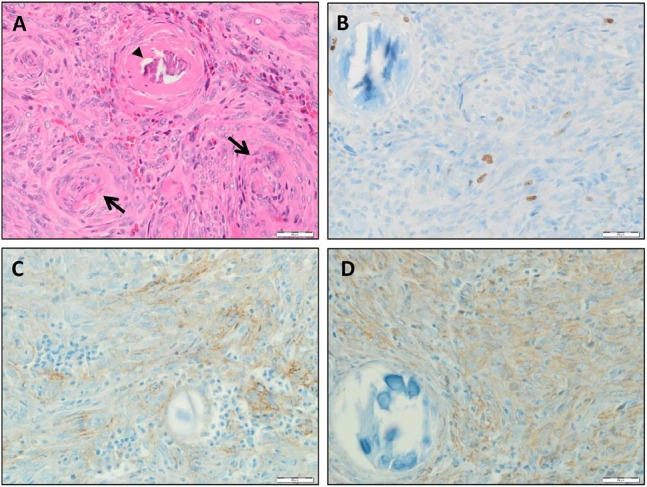
Histological features of meningioma in Cowden syndrome **A.** Histologic examination presented a meningioma characterized by onion bulb formations (→) and psamomma bodies (►) which both are characteristics for meningioma histopathology. **B.** Immunocytological confirmation of meningioma tumor by Mib1/Ki67 staining. Note the positive cells (brown dye) next to psamomma body. **C.** Immunopositivity was detected for EMA. **D.** Immunopositive Vimentin stained cells are detecable. Scale bar represents 20 μm.

Further, we closely followed up the case of a 29-year-old female who was referred to our hospital for the evaluation of her cervical polyps. A diagnostic hysteroscopy with fractional curettage was performed revealing an endometrial carcinoma (grade II). The patient underwent a radical hysterectomy. Postoperative therapy included brachytherapy in afterload technique in the vagina. Approximately 12 months later after a routine clinical examination, she displayed a nodular goiter. The patient underwent a subtotal thyroidectomy. Due to unclear intestinal complaints, a colonoscopy was performed. In an endoscopic examination small polyps were found in the sigmoid colon and rectum from which a biopsy was taken (Figure 1). Histopathological investigations revealed the overall picture of a mucosal ganglioneuroma. The neurological examination showed significant dysdiadochokinesia on both sides, as well as an ataxic uncertain gait pattern. Cerebral magnetic resonance imaging (MRI) was performed for further evaluation. MRI scans revealed two infra-tentorial tumorous lesions on the left side (Figure 2). Due to tumor increase, a resection was performed, and the histopathological examination of the excised tumor tissue showed meningioma WHO° I (Figure 3). Physical examination revealed macrocephaly, multiple facial papules, gingival fitrichilemmomas and acral keratoses. The family history was inconspicuous concerning CS and neurological syndromes.

The patient presented clinical signs all indicative for the Cowden syndrome (Table 2). Subsequently, human genetic test detected a novel germline G132F mutation in PTEN gene. This mutation occurs within exon 5, which encodes a portion of the phosphatase domain. Interestingly, the G132F missense mutation has not previously been reported in association with Cowden syndrome.

Finally, we summarized 109 cases of CS from the peer-reviewed literature featured in Supplementary Table S1.

## DISCUSSION

The main purpose of this paper was to investigate the occurrence of meningiomas in patients with Cowden syndome. A particular attention is paid to the role of PTEN mutation in the complex syndrome-related diseases [10]. In fact follows a PTEN inactivation hyperactivation of the PI3K-Akt signaling pathway in many human tumors, including meningioma. Moreover, PTEN mutations predispose to Cowden syndrome. Our results verified for the first time the hidden association of Cowden syndrome with PTEN mutation and meningiomas.

Our observations that brain tumors, including gangliocytoma, are frequently present in Cowden syndrome patients are not novel. However, this study provides strong evidence for a high incidence of meningiomas in patients with Cowden syndrome (gangliocytoma 9%, meningioma 8%). Our data show that approximately 41% of the brain tumor cases (n=22) were meningiomas. This finding is further supported by the sex distribution of meningiomas in CS patients: In fact the female to male ratio of menigiomas in CS patients was 4:5, whereas generally meningiomas show a sex distribution of 2.1 : 1 (female to male). This sex distribution does not follow the normal distribution of mengiomas and thus is another independent evidence for the specific association of meningiomas in CS.

An important implication of these findings is that meningiomas should be taken under consideration by monitoring of patients with Cowden syndrome. Thus, we propose to include neurological diagnostic criteria for encompassing all clinical features of Cowden syndrome.

Although meningiomas are frequent, it is still uncertain whether one part of the PTEN gene sequence accounts for meningioma or whether multiple mutations in various genes cause this tumor entity.

**Figure 4:**
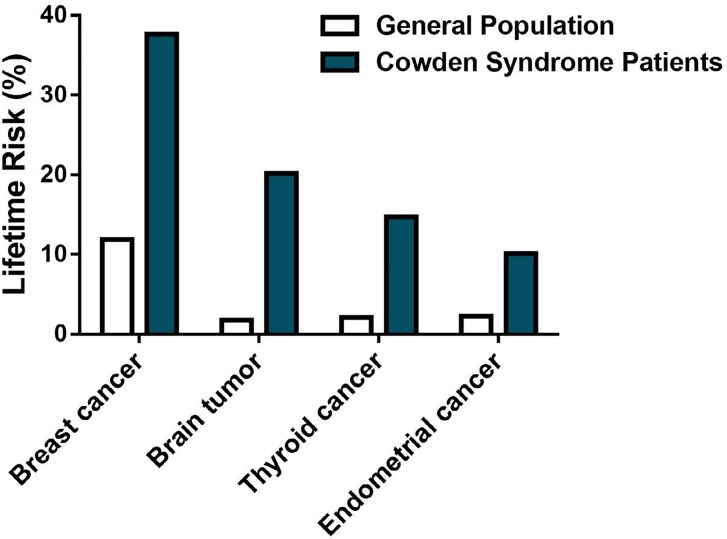
Lifetime risk of selected tumors in Cowden syndrome Summary of 109 reported cases diagnosed with Cowden syndrome. Patients suffering from Cowden syndrome, a rare inherited disease characterized in part by benign neoplasia growth throughout the body, are at high risk of several tumors, including breast cancer (37% lifetime risk), brain tumor (20% lifetime risk), thyroid cancer (14% lifetime risk), and endometrial cancer (10% lifetime risk).

The association between Cowden syndrome, PTEN mutation, and meningiomas is of clinical relevance. The universal use of non-invasive brain imaging techniques such as cerebral computerized tomography (C-CT) and magnetic resonance imaging (MRI) can dramatically improve the diagnostic ability of this type of tumor. Early diagnosis and close monitoring of patients with Cowden syndrome is important in view of the malignancies associated with this rare disease. Considering that 20% of Cowden syndrome patients tend to develop brain tumors (Figure 4), further investigations on the relation between Cowden syndrome, PTEN mutation, and meningiomas is warranted. The presented data from our cohort investigation give good evidence to include brain tumor monitoring to the criteria of the International Cowden Consortium and screening recommendations of the National Comprehensive Cancer Network (Table 2). Thus we propose to consider meningiomas in patients with CS and other PTEN-related diseases such as PTEN Hamartoma tumor syndrome.

## MATERIALS AND METHODS

A systematic review of published results was performed by the authors using PubMed (http://www.ncbi.nlm.nih.gov/pubmed). The search terms used were Cowden syndrome, Cowden disease, multiple hamartoma syndrome, PTEN, MMAC1, meningioma, PTEN hamartoma tumor syndrome (PHTS). We only used reports from 1995 to 2016 and excluded cases with patients less than 18 years of age or non-English written reports. Cases were included when Cowden syndrome patients and PTEN mutation and tumor were reported. Cases that included patients without examination of the central nervous system were excluded. The data collected was as follows: age, gender, PTEN mutation, MRI examination, breast tumor, thyreoid tumor, endometrial tumor, renal cancer, colorectal cancer, and brain tumors.

## SUPPLEMENTARY DATA TABLE



## References

[1] Eng C (2000). Will the real Cowden syndrome please stand up: revised diagnostic criteria. J Med Genet.

[2] Masmoudi A, Chermi ZM, Marrekchi S, Raida BS, Boudaya S, Mseddi M, Jalel MT, Turki H (2011). Cowden syndrome. J Dermatol Case Rep.

[3] Scheper MA, Nikitakis NG, Sarlani E, Sauk JJ, Meiller TF (2006). Cowden syndrome: report of a case with immunohistochemical analysis and review of the literature. Oral Surg Oral Med Oral Pathol Oral Radiol.

[4] Li J, Yen C, Liaw D, Podsypanina K, Bose S, Wang SI, Puc J, Miliaresis C, Rodgers L, McCombie R, Bigner SH, Giovanella BC, Ittmann M (1997). PTEN, a putative protein tyrosine phosphatase gene mutated in human brain, breast, and prostate cancer. Science.

[5] Steck PA, Pershouse MA, Jasser SA, Yung WK, Lin H, Ligon AH, Langford LA, Baumgard ML, Hattier T, Davis T, Frye C, Hu R, Swedlund B (1997). Identification of a candidate tumour suppressor gene, MMAC1, at chromosome 10q23.3 that is mutated in multiple advanced cancers. Nature Genet.

[6] Nelen MR, van Staveren WC, Peeters EA, Hassel MB, Gorlin RJ, Hamm H, Lindboe CF, Fryns JP, Sijmons RH, Woods DG, Mariman EC, Padberg GW, Kremer H (1997). Germline mutations in the PTEN/MMAC1 gene in patients with Cowden disease. Hum Mol Genet.

[7] Starink TM, van der Veen JP, Arwert F, de Waal LP, de Lange GG, Gille JJ, Eriksson AW (1986). The Cowden syndrome: a clinical and genetic study in 21 patients. Clin Genet.

[8] Nelen MR, Padberg GW, Peeters EA, Lin AY, van den Helm B, Frants RR, Coulon V, Goldstein AM, van Reen MM, Easton DF, Eeles RA, Hodgsen S, Mulvihill JJ (1996). Localization of the gene for Cowden disease to chromosome 10q22-23. Nat Genet.

[9] Marsh DJ, Coulon V, Lunetta KL, Rocca-Serra P, Dahia PL, Zheng Z, Liaw D, Caron S, Duboue B, Lin AY, Richardson AL, Bonnetblanc JM, Bressieux JM (1998). Mutation spectrum and genotype-phenotype analyses in Cowden disease and Bannayan-Zonana syndrome, two hamartoma syndromes with germline PTEN mutation. Hum Mol Gen.

[10] Eng C (2003). PTEN: one gene, many syndromes. Human mutat.

[11] Daly MB, Pilarski R, Axilbund JE, Buys SS, Crawford B, Friedman S, Garber JE, Horton C, Kaklamani V, Klein C, Kohlmann W, Kurian A, Litton J (2014). National comprehensive cancer n. Genetic/familial high-risk assessment: breast and ovarian, version 1.2014. J Natl Compr Canc Netw.

[12] Dobbins SE, Broderick P, Melin B, Feychting M, Johansen C, Andersson U, Brannstrom T, Schramm J, Olver B, Lloyd A, Ma YP, Hosking FJ, Lonn S (2011). Common variation at 10p12.31 near MLLT10 influences meningioma risk. Nat Genet.

[13] Rempel SA, Schwechheimer K, Davis RL, Cavenee WK, Rosenblum ML (1993). Loss of heterozygosity for loci on chromosome 10 is associated with morphologically malignant meningioma progression. Cancer Res.

[14] Mihaila D, Jankowski M, Gutierrez JA, Rosenblum ML, Newsham IF, Bogler O, Rempel SA, Consortium NC (2003). Meningiomas: loss of heterozygosity on chromosome 10 and marker-specific correlations with grade, recurrence, and survival. Clin Cancer Res.

[15] Mihaila D, Gutierrez JA, Rosenblum ML, Newsham IF, Bogler O, Rempel SA, Consortium NC (2003). Meningiomas: analysis of loss of heterozygosity on chromosome 10 in tumor progression and the delineation of four regions of chromosomal deletion in common with other cancers. Clin Cancer Res.

[16] Weber RG, Bostrom J, Wolter M, Baudis M, Collins VP, Reifenberger G, Lichter P (1997). Analysis of genomic alterations in benign, atypical, and anaplastic meningiomas: toward a genetic model of meningioma progression. Proc Natl Acad Sci U S A.

[17] Simon M, von Deimling A, Larson JJ, Wellenreuther R, Kaskel P, Waha A, Warnick RE, Tew JM, Menon AG (1995). Allelic losses on chromosomes 14, 10, and 1 in atypical and malignant meningiomas: a genetic model of meningioma progression. Cancer Res.

