# Supplementary material for: Hidden association of Cowden syndrome, PTEN mutation and meningioma frequency
Source: Oncoscience. 2016 Jun 30;3(5-6):149–55. doi: 10.18632/oncoscience.305 (PMC4965257; doi:10.18632/oncoscience.305)
Supplement: Supplementary file 1 [file oncoscience-03-149-s001.pdf]

SUPPLEMENTARY DATA

REFERENCES

1. Busch RM, Chapin JS, Mester J, Ferguson L, Haut JS, Frazier TW, Eng C. Cognitive characteristics of PTEN hamartoma tumor syndromes. Genet Med. 2013; 15: 548-553.
2. Tsou HC, Ping XL, Xie XX, Gruener AC, Zhang H, Nini R, Swisshelm K, Sybert V, Diamond TM, Sutphen R, Peacocke M. The genetic basis of Cowden's syndrome: three novel mutations in PTEN/MMAC1/TEP1. Hum Genet. 1998; 102: 467-473.
3. Liaw D, Marsh DJ, Li J, Dahia PL, Wang SI, Zheng Z, Bose S, Call KM, Tsou HC, Peacocke M, Eng C, Parsons R. Germline mutations of the PTEN gene in Cowden disease, an inherited breast and thyroid cancer syndrome. Nat Genet. 1997; 16:64-67.
4. Lindboe CF, Helseth E, Myhr G. Lhermitte-Duclos disease and giant meningioma as manifestations of Cowden's disease. Clin Neuropathol. 1995; 14: 327-330.
5. Nelen MR, van Staveren WC, Peeters EA, Hassel MB, Gorlin RJ, Hamm H, Lindboe CF, Fryns JP, Sijmons RH, Woods DG, Mariman EC, Padberg GW, Kremer H. Germline mutations in the PTEN/MMAC1 gene in patients with Cowden disease. Hum Mol Genet. 1997; 6: 1383-1387.
6. Winter H, McEwen A. Cowden syndrome: presenting as advanced breast cancer in a young woman with macrocephaly. Intern Med J. 2012; 42: 1160-1161.
7. Kersseboom R, Dubbink HJ, Corver WE, van Tilburg AJ, Poley JW, van Leerdam ME, Atmodimedjo PN, van de Laar IM, Collee JM, Dinjens WN, Morreau H, Wagner A. PTEN in colorectal cancer: a report on two Cowden syndrome patients. Clin Genet. 2012; 81: 555-562.
8. Lok C, Viseux V, Avril MF, Richard MA, Gondry-Jouet C, Deramond H, Desfossez-Tribout C, Courtade S, Delaunay M, Piette F, Legars D, Dreno B, Saiag P, et al. Cancerology Group of the French Society of D. Brain magnetic resonance imaging in patients with Cowden syndrome. Medicine. 2005; 84: 129-136.
9. Nelen MR, Kremer H, Konings IB, Schoute F, van Essen AJ, Koch R, Woods CG, Fryns JP, Hamel B, Hoefsloot LH, Peeters EA, Padberg GW. Novel PTEN mutations in patients with Cowden disease: absence of clear genotype-phenotype correlations. Eur J Hum Genet. 1999; 7: 267-273.
10. Celebi JT, Tsou HC, Chen FF, Zhang H, Ping XL, Lebwohl MG, Kezis J, Peacocke M. Phenotypic findings of Cowden syndrome and Bannayan-Zonana syndrome in a family associated with a single germline mutation in PTEN. J Med Genet. 1999; 36: 360-364.
11. Celebi JT, Ping XL, Zhang H, Remington T, Sulica VI, Tsou HC, Peacocke M. Germline PTEN mutations in three families with Cowden syndrome. Exp Dermatol. 2000; 9: 152-156.
12. Tate G, Suzuki T, Endo Y, Mitsuya T. A novel mutation of the PTEN gene in a Japanese patient with Cowden syndrome and bilateral breast cancer. Cancer Genet Cytogenet. 2008; 184: 67-71.
13. Sawada T, Okada T, Miwa K, Satoh H, Asano A, Mabuchi H. Two novel mutations of PTEN gene in Japanese patients with Cowden syndrome. Am J Med Genet. 2004; 128A: 12-14.
14. Sawada T, Hamano N, Satoh H, Okada T, Takeda Y, Mabuchi H. Mutation analysis of the PTEN / MMAC1 gene in Japanese patients with Cowden disease. Jpn J Cancer Res. 2000; 91: 700-705.
15. Vega A, Torres J, Torres M, Cameselle-Teijeiro J, Macia M, Carracedo A, Pulido R. A novel loss-of-function mutation (N48K) in the PTEN gene in a Spanish patient with Cowden disease. J Invest Dermatol. 2003; 121: 1356-1359.
16. Zbuk KM, Patocs A, Shealy A, Sylvester H, Miesfeldt S, Eng C. Germline mutations in PTEN and SDHC in a woman with epithelial thyroid cancer and carotid paraganglioma. Nat Clin Pract Oncol. 2007; 4: 608-612.
17. Loffeld A, McLellan NJ, Cole T, Payne SJ, Fricker D, Moss C. Epidermal naevus in Proteus syndrome showing loss of heterozygosity for an inherited PTEN mutation. Br J Dermatol. 2006; 154: 1194-1198.
18. Marsh DJ, Dahia PL, Caron S, Kum JB, Frayling IM, Tomlinson IP, Hughes KS, Eeles RA, Hodgson SV, Murday VA, Houlston R, Eng C. Germline PTEN mutations in Cowden syndrome-like families. J Med Genet. 1998; 35: 881-885.
19. Marsh DJ, Coulon V, Lunetta KL, Rocca-Serra P, Dahia PL, Zheng Z, Liaw D, Caron S, Duboue B, Lin AY, Richardson AL, Bonnetblanc JM, Bressieux JM, et al. Mutation spectrum and genotype-phenotype analyses in Cowden disease and Bannayan-Zonana syndrome, two hamartoma syndromes with germline PTEN mutation. Hum Mol Genet. 1998; 7: 507-515.
20. Kubo Y, Urano Y, Hida Y, Ikeuchi T, Nomoto M, Kunitomo K, Arase S. A novel PTEN mutation in a Japanese patient with Cowden disease. Br J Dermatol. 2000; 142: 1100-1105.
21. Staal FJ, van der Luijt RB, Baert MR, van Drunen J, van Bakel H, Peters E, de Valk I, van Amstel HK, Taphoorn MJ, Jansen GH, van Veelen CW, Burgering B, Staal GE. A novel germline mutation of PTEN associated with brain tumours of multiple lineages. Br J Cancer. 2002; 86: 1586-1591.
22. Kohno T, Takahashi M, Fukutomi T, Ushio K, Yokota J. Germline mutations of the PTEN/MMAC1 gene in Japanese patients with Cowden disease. Jpn J Cancer Res. 1998; 89: 471-474.

23. Bussaglia E, Pujol RM, Gil MJ, Marti RM, Tuneu A, Febrer MI, Garcia-Patos V, Ruiz EM, Barnadas M, Alegre M, Serrano S, Matias-Guiu X. PTEN mutations in eight Spanish families and one Brazilian family with Cowden syndrome. J Invest Dermatol. 2002; 118: 639-644.
24. Iida S, Nakamura Y, Fujii H, Kimura M, Moriwaki K. A heterozygous germline mutation of the PTEN/MMAC1 gene in a patient with Cowden disease. Int J Mol Med. 1998; 1: 565-568.
25. Kurose K, Araki T, Matsunaka T, Takada Y, Emi M. Variant manifestation of Cowden disease in Japan: hamartomatous polyposis of the digestive tract with mutation of the PTEN gene. Am J Hum Genet. 1999; 64: 308-310.
26. Negoro K, Takahashi S, Kinouchi Y, Takagi S, Hiwatashi N, Ichinohasama R, Shimosegawa T, Toyota T. Analysis of the PTEN gene mutation in polyposis syndromes and sporadic gastrointestinal tumors in Japanese patients. Dis Colon Rectum. 2000; 43: 29-33.
27. Kato N, Kimura K, Sugawara H, Aoyagi S, Ikeda T, Horii A. Germline mutation of the PTEN gene in a Japanese patient with Cowden's disease. Int J Oncol. 2001; 18: 1017-1022.
28. Derrey S, Proust F, Debono B, Langlois O, Layet A, Layet V, Longy M, Freger P, Laquerriere A. Association between Cowden syndrome and Lhermitte-Duclos disease: report of two cases and review of the literature. Surg Neurol. 2004; 61: 447-454.
29. Lynch ED, Ostermeyer EA, Lee MK, Arena JF, Ji H, Dann J, Swisshelm K, Suchard D, MacLeod PM, Kvinnsland S, Gjertsen BT, Heimdal K, Lubs H, et al. Inherited mutations in PTEN that are associated with breast cancer, cowden disease, and juvenile polyposis. Am J Hum Genet. 1997; 61: 1254-1260.
30. Salo-Mullen EE, Shia J, Brownell I, Allen P, Girotra M, Robson ME, Offit K, Guillem JG, Markowitz AJ, Stadler ZK. Mosaic partial deletion of the PTEN gene in a patient with Cowden syndrome. Fam Cancer. 2014; 13: 459-467.
31. Heindl M, Handel N, Ngeow J, Kionke J, Wittekind C, Kamprad M, Rensing-Ehl A, Ehl S, Reifenberger J, Loddenkemper C, Maul J, Hoffmeister A, Aretz S, et al. Autoimmunity, intestinal lymphoid hyperplasia, and defects in mucosal B-cell homeostasis in patients with PTEN hamartoma tumor syndrome. Gastroenterology. 2012; 142: 1093-1096.
32. Gicquel JJ, Vabres P, Bonneau D, Mercie M, Handiri L, Dighiero P. Retinal angioma in a patient with Cowden disease. Am J Ophthalmol. 2003; 135: 400-402.
33. Sanson M, Zhou XP, Brault JL, Hoang-Xuan K, Hamelin R. A novel germline mutation of the PTEN/MMAC1 gene in a patient with Cowden disease. Acta Oncol. 1999; 38: 973-975.

Supplementary Table S1: Clinical features of the 109 patients with Cowden syndrome all carrying PTEN mutation

Case	Age	Sex	PTEN Genotype/ PTEN Mutation	Brain MRI Findings	Breast	Thyroid	Renal Cancer	Endometrium	Colorectal Cancer	Brain Tumor	Reference
1	60	M	c.395G>A p.(Gly132Asp)	Mn		b	Y			Y	[1]
2	54	F	c.388C>T p.(Arg130Ter)	Mn	50,52m b	b		b		Y	[1]
3	23	M	c.382A>G p.(Lys128Glu)	CH		b				N	[1]
4	45	F	c.697C>T p.(Arg233Ter)	LD	44m b	42m b				Y	[1]
5	44	F	c.734_737del4		b	41m b		38m		N	[1]
6	36	F	c.865_871delAAAGTAG	V	b	36m b				N	[1]
7	50	M	c.253+1G>A	LD		44m b				Y	[1]
8	51	F	Exon 2 deletion		44m b	b				N	[1]
9	29	F	c.394GG>TT p.(Gly132Phe)	Mn	b	b		29m		Y	PS
10	26	F	c.335T>C p.(Leu112Pro)	MnLD	b	b		b		Y	[2]
11	75	F	c.202T>C p.(Tyr68His)		b	b				N	[2]
12		M	c.386G>A p.(Gly129Glu)	GB	b					Y	[3]
13			c.386G>A p.(Gly129Glu)			b				N	[3]
14	43	M	c.469G>T p.(Glu157Ter)	LD Mn						Y	[4]
15	57	M	c.368A>G p.(His123Arg)			b				N	[5]
16	42	F	c.370T>C p.(Cys124Arg)			b				N	[5]
17	38	F	c.387C>T p.(Arg130Ter)		b	b				N	[5]
18	35	F	c.15delC		35m	b				N	[6]
19	28	M	p.(His123Gln)			b			Y	N	[7]
20	39	M	c.634+5G>A			b			Y	N	[7]
21	50	M	Y	Mn		b				Y	[8]
22	38	M	Y	LD		b				Y	[8]
23	34	M	Y	V		b				N	[8]
24	56	M	Y	V		b				N	[8]
25	58	F	Y	LD V	b			m		Y	[8]
26	36	F	Y		m					N	[8]

(Continued)

Case	Age	Sex	PTEN Genotype/ PTEN Mutation	Brain MRI Findings	Breast	Thyroid	Renal Cancer	Endometrium	Colorectal Cancer	Brain Tumor	Reference
27	26	F	Y		m	m				N	[8]
28	72	M	Y							N	[8]
29	36	F	Y		m			b		N	[8]
30	50	F	Y		m			m		N	[8]
31	48	M	Y			m			Y	N	[8]
32	48	F	Y		m<br>b					N	[8]
33	32	F	Y							N	[8]
34	41	F	c.389G>A p.(Arg130Gln)		29m<br>b	b		b		N	[1]
35	30	F	c.287C>G p.(Pro96Arg)		b	b				N	[1]
36	44	F	c.634+1G>C		43m<br>b	b	44Y			N	[1]
37	58	F	c.697C>T p.(Arg233Ter)		56m<br>b	b		53m	34Y	N	[1]
38	58	F	c.1033C>G p.(Leu345Val)		46m<br>b	b		41m<br>b		N	[1]
39	46	F	c.401T>C p.(Met134Thr)		43m<br>b	46m<br>b		b		N	[1]
40	58	F	Whole gene deletion		56m<br>b	b		b		N	[1]
41	25	F	c.253+1G>C		b	b				N	[1]
42	57	M	c.253+1G>C			b				N	[1]
43	26	F	c.486_487delCA		b	b				N	[1]
44		F	p.(Gly165Glu)		b	m<br>b				N	[9]
45	31	F	c.1003C>T p.(Arg335Ter)	MS	21m	10m				N	[10]
46	54	F	c.1003C>T p.(Arg335Ter)		48m	42b				N	[10]
47	47	F	c.106G>A p.(Gly36Arg)		28,31m	b		b		N	[11]
48	43	F	c.388C>T p.(Arg130Ter)		b	b				N	[11]
49	51	M	c.375insTTTA			b				N	[11]
50	50	F	c.115G>C p.(Arg39Pro)		m<br>b	b		b		N	[12]
51	41	M	c.697C>T p.(Arg233Ter)	Mn		b				Y	[13]
52	70	F	c.589A>T p.(Lys197Ter)			b				N	[13]

(*Continued*)

Case	Age	Sex	PTEN Genotype/ PTEN Mutation	Brain MRI Findings	Breast	Thyroid	Renal Cancer	Endometrium	Colorectal Cancer	Brain Tumor	Reference
53	39	M	c.219-222delAAAGA			b				N	[13]
54	48	F	c.1004G>T p.(Arg335Leu)		m	b				N	[14]
55	38	F	c.632insG			b				N	[14]
56	40	M	I3+1delGT							N	[14]
57	62	F	I3+1delGTAA		m	m				N	[14]
58	28	M	c.697C>T p.(Arg233Ter)		b	b				N	[14]
59	38	F	p.(Asn48Lys)		m	b		m		N	[15]
60	43	F	c.48-48insA		b	m		b		N	[16]
61		F	p.(Tyr68Asp)							N	[17]
62	31	M	c.209T>C p.(Leu70Pro)			m				N	[18]
63	75	F	p.(Tyr68His)		b	b				N	[19]
64		F	p.(Arg130Leu)		m<br>b	m<br>b			Y	N	[19]
65		F	p.(Gly165Val)	Mn	b	b				Y	[19]
66	57	F	c.1003C>T p.(Arg335Ter)		43,51m					N	[20]
67	55	F	c.388C>T p.(Arg130Ter)		48m	18b		37m<br>b	Y	N	[20]
68	30	F	c.406T>C p.(Cys136Arg)		19b	18b			Y	N	[20]
69	38	M	c.701G>A p.(Arg234Gln) ?	OD<br>Mn						Y	[21]
70	56	F	c.530-531delAT		53m	b				N	[22]
71	49	M	c.247insT							N	[22]
72	46	M	c.277C>T p.(His93Tyr)			b				N	[22]
73	72	M	p.(Pro96Gln)			b				N	[23]
74	62	F	c.528T>G p.(Tyr176Ter)		m	b				N	[23]
75	53	F	p.(His123Asp)			b				N	[23]
76	32	F	p.(Arg335Ter)	LD	b	b				Y	[23]
77	45	F	p.(Gln214Ter)		m<br>b	m				N	[23]
78	38	F	c.487-491delA		b					N	[23]
79	36	M	p.(Arg233Ter)			b				N	[23]
80			c.388C>T p.(Arg130Ter)							N	[24]
81	35	M	c.389G>A p.(Arg130Gln)			b				N	[25]

(*Continued*)

Case	Age	Sex	PTEN Genotype/ PTEN Mutation	Brain MRI Findings	Breast	Thyroid	Renal Cancer	Endometrium	Colorectal Cancer	Brain Tumor	Reference
82	54	F	I9-1A>G	LD	b	b				Y	[26]
83	56	F	c.388C>T p.(Arg130Ter)		m	b			Y	N	[27]
84	25	F	c.359T>G Stopp codon	LD		b				Y	[28]
85	27	F	c.395G>A p.(Gly132Ter)	LD		b				Y	[28]
86	46	F	c.68T>A p.(Leu22Ter)		46m					N	[29]
87	24	F	c.68T>A p.(Leu22Ter)		24m	18m				N	[29]
88	34	F	c.328C>T p.(Gln109Ter)		34m					N	[29]
89	31	F	c.565A>T p.(Arg188Ter)	NF	31m	b				Y	[29]
90	29	F	c.565A>T p.(Arg188Ter)		29b					N	[29]
91	27	F	c.697C>T p.(Arg232Ter)		27m					N	[29]
92	42	F	c.1003C>T p.(Arg334Ter)		41b	42b		42m	42Y	N	[29]
93	50	F	c.1028T>A p.(Val332Glu) +c.1039T>C p.(Phe346Leu)	NE	50m	49b				Y	[29]
94	48	F	c.1028T>A p.(Val332Glu) +c.1039T>C p.(Phe346Leu)		30b	32b		48m		N	[29]
95	42	F	c.1028T>A p.(Val332Glu) +c.1039T>C p.(Phe346Leu)		33b	33b		42m		N	[29]
96		M	c.1028T>A p.(Val332Glu) +c.1039T>C p.(Phe346Leu)	PN						Y	[29]
97	42	F	c.1028T>A p.(Val332Glu) +c.1039T>C p.(Phe346Leu)		42m					N	[29]
98	43	F	exons 6-9del		m b	b		b		N	[30]
99	56	M	c.534T>G p.(Tyr178Ter)			m				N	[31]
100	54	F	c.334C>G p.(Leu112_Lys164del)		m b					N	[31]
101	37	M	c.388C>TSNP c.1026+32T>G p.(Arg130Ter)			b			Y	N	[31]
102	53	M	c.395G>A p.(Gly132Asp)			b	Y			N	[31]
103	53	M	c.80-?_164+?del			b				N	[31]
104	44	F	c.49C>T p.(Gln17Ter)		b	b				N	[31]
105	38	M	c.634+2T>C							N	[31]

(*Continued*)

Case	Age	Sex	PTEN Genotype/ PTEN Mutation	Brain MRI Findings	Breast	Thyroid	Renal Cancer	Endometrium	Colorectal Cancer	Brain Tumor	Reference
106	48	F	c.389G>A p.(Arg130Gln)		m<br>b			b		N	[31]
107	37	M	c.376G>C p.(Ala126Pro)			b				N	[31]
108	50	F	c.464A>G p.(Tyr155Cys)		m	b				N	[32]
109	48	F	p.(Gln214Ter)	Me	30m	44b	Y			Y	[33]

Abbreviations used: (b) benign; (C) Cystic lesion; (CH) Chiari I Malformation; (F) female; (GB) Glioblastoma; (LD) Dysplastic gangliocytoma; (m) malignant; (M) male; (Me) Metastases; (Mn) Meningioma; (MS) Multiple sclerosis; (NE) Malignant neuroma; (N) absent; (NF) Neurofibroma; (OD) Oligodendroglioma; (PN) Pineal-gland tumor; (PS) present study; (V) Venous anomaly/angioma; (Y) present.
